# Effect of Cognitive Variables on the Reading Ability of Spanish Children at Age Seven

**DOI:** 10.3389/fpsyg.2021.663596

**Published:** 2021-05-10

**Authors:** María José González-Valenzuela, Dolores López-Montiel, Félix Díaz-Giráldez, Isaías Martín-Ruiz

**Affiliations:** ^1^Departamento de Psicología Evolutiva y de la Educación, Facultad de Psicología, Universidad de Málaga, Málaga, Spain; ^2^Departamento de Psicobiología y Metodología de las Ciencias del Comportamiento, Facultad de Psicología, Universidad de Málaga, Málaga, Spain

**Keywords:** naming speed, phonological memory, reading words, primary education, phonological awareness, knowledge of letters

## Abstract

The objective of this study is to determine the contribution made by knowledge of letters, phonological awareness, phonological memory, and alphanumeric and non-alphanumeric rapid automatized naming at the ages of six and seven to the ability of Spanish children to read words at 7 years of age. A total of 116 Spanish-speaking school children took part in the study, from schools located in an average socio-cultural setting, without special educational needs. The reading ability of these pupils was evaluated at the age of seven, and cognitive variables were assessed at 6 and 7 years of age. Descriptive-exploratory analyses, bivariate analyses, and multivariate regressions were performed. The results show that cognitive variables measured at these ages contribute differently to the ability to read words at 7 years of age. Rapid naming does not seem to influence word reading; knowledge of letters no longer influences word reading as children grow older; and phonological awareness and phonological memory maintain their contribution to the explanation of word reading. These results indicate that reading in Spanish depends on different cognitive variables and that this relationship varies according to age. The findings have key educational implications in terms of teaching reading skills and the prevention of specific learning difficulties in Spanish Primary Education.

## Introduction

In recent years, an important strand of research has been established on the different variables that determine the acquisition of reading skill. Numerous studies have found diverse relationships between different cognitive variables and learning to read in languages of different linguistic consistency and at different ages (Li et al., 2012; Caravolas et al., 2013; Asadi et al., 2017; López-Escribano et al., 2018; Mcilraith, 2018; Bar-Kochva and Nevo, 2019; Torppa et al., 2019; Vibulpatanavong and Evans, 2019; Wijaythilake et al., 2019). Most of these studies have highlighted different relationships between knowledge of letters, phonological awareness, rapid automatized naming, and phonological memory, among other variables, and reading, according to different reading variables considered, the age of the pupil and the linguistic consistency of the language.

It should be noted that there does not seem to be a consensus about whether the role that these variables play in explaining reading at different ages is similar or different depending on the orthographic consistency, this being one of the reasons for studying the predictors in Spanish. There is research which supports that the relationship between some of these variable and reading seems to be similar in languages of different consistency according to age and they believe in a universal theory of reading acquisition (Frost, 2005; Ziegler and Goswami, 2005; Furnes and Samuelsson, 2009; Caravolas et al., 2013). On the other hand, some research supports the variability of the influence of these variables on reading depending on the orthographic consistency and age (Furnes and Samuelsson, 2009; Vaessen et al., 2010; Ziegler et al., 2010; Tibi and Kirby, 2017; Landerl et al., 2019). In addition, the theory of granularity supports the idea that the degree of consistency is relative, since a language will be less orthographic consistent when graphemes represent the phonemes in a less precise way due to their phonological structure (coarse grain), whereas it will be more consistent when graphemes represent more precisely the phonemes (fine grain) (Ziegler and Goswami, 2005). This could justify the predictors of reading to be different depending the grain of each language and the interest of our research.

In the case of Spanish, there are far more studies on the predictive factors of reading at an early age (Casillas and Goikoetxea, 2007; Aguilar-Villagrán et al., 2010; González-Seíjas et al., 2013; Suárez-Coalla et al., 2013; De la Calle-Cabrera et al., 2019), with far fewer conducted at older ages, when learning difficulties begin to be diagnosed. However, the analysis of predictors of reading at older ages might be of interest in order to detect and explain the learning disabilities. It should also be noted that studies carried out in Spanish when children are literate often show different results, since they use a variety of reading measures (precision, efficiency, or fluency) and other predictors related not only to reading precision and efficiency, but also to reading comprehension. Moreover, these studies do not distinguish between components of rapid automatized naming, and they use different measures to evaluate phonological awareness, which is another of the reasons for the objective of this study.

Knowledge of letters has a strong influence on reading in different languages and at different ages (Casillas and Goikoetxea, 2007; Burke et al., 2009; Furnes and Samuelsson, 2009; Caravolas et al., 2012, 2013; Georgiou et al., 2012; Tolchinsky et al., 2012; Solari et al., 2014; Mcilraith, 2018; Wijaythilake et al., 2019), since it favors grapheme-phoneme conversion in the reading process, especially in consistent languages. Some of these studies highlight its relationship with reading words and pseudowords in early years education (Burke et al., 2009; Caravolas et al., 2012, 2013; Georgiou et al., 2012; López-Escribano et al., 2018) and in primary education (Casillas and Goikoetxea, 2007; Caravolas et al., 2012, 2013; Georgiou et al., 2012; De la Calle-Cabrera et al., 2019). However, other studies have only detected an influence at younger ages (Furnes and Samuelsson, 2009; Tolchinsky et al., 2012; Holopainen et al., 2016) and not among older children (Landerl et al., 2013; Solari et al., 2014), since knowledge of letters would already be acquired at these ages, and other types of cognitive-linguistic variables would become more relevant (Paige et al., 2018). Given this controversy and the fact that, in Spanish, most studies tend to focus on this aspect in early years education, we decided to include this variable in our study.

The role of phonological awareness in reading in different languages and at different ages is also controversial. Some studies on consistent languages have found that phonological awareness predicts more reading accuracy than fluency and that it may no longer be an important predictor of reading at older ages (Babayigit and Stainthorp, 2011). In turn, there are studies that prove the phonological awareness to predict reading accuracy for a longer period in opaque languages than in transparent languages (Landerl et al., 2019). This might be due to the fact that reading fluency carries with it a measure of speed as opposed to reading precision, being the phonological awareness influential only when it comes to performing tasks of precision rather than speed (Babayigit and Stainthorp, 2011; Landerl et al., 2019). Nevertheless, other studies have shown that phonological awareness predicts both reading fluency and accuracy, and that it continues to be a good predictor in consistent languages and at older ages, due to the importance of the grapheme-phoneme correspondence in reading processes (Casillas and Goikoetxea, 2007; Furnes and Samuelsson, 2010; González-Trujillo et al., 2014; González-Valenzuela et al., 2016; Bar-Kochva and Nevo, 2019). On the other hand, some studies indicate that phonological awareness might be important only during the first years of schooling in consistent languages (Leppänen et al., 2006; Pittas, 2018) and during subsequent years in inconsistent languages (Nielsen and Juul, 2016; Vibulpatanavong and Evans, 2019). That is, the role of phonological awareness in reading depends on the orthographic consistency, the reading variable considered and the age of the students (González-Valenzuela et al., 2016). Given this controversy, in this research it has been considered the contribution of the phonological awareness in Spanish students at the age of 6 and 7 to the reading fluency at the age of 7. The purpose of this is to check whether in Spanish the role of phonological awareness is important after literacy in this reading measure, and if it fades with age as occurs in other consistent languages or in other reading measures. The measure of reading fluency has been considered because the children at the age of seven are already literate in Spanish, and in addition to having learned by then to recognize letters with accuracy, they have already automated the recognition process and they do so with speed.

Rapid automatized naming, defined as the ability to quickly name visual items, is shown to be a predictor of reading in different orthographic consistency languages (Aguilar-Villagrán et al., 2010; Furnes and Samuelsson, 2010; Georgiou et al., 2012, 2016; Ferroni et al., 2016; González-Valenzuela et al., 2016; López-Escribano et al., 2018; Bar-Kochva and Nevo, 2019; De la Calle-Cabrera et al., 2019; Landerl et al., 2019). In consistent orthographies, the reader decodes by applying the grapheme-phoneme conversion rules, and the phonological representation of each grapheme must be recovered quickly for the grapheme-phoneme recoding strategy to be effective. For this reason, rapid automatized naming is closely related to reading, especially with reading speed and fluency, being a better predictor than the phonological awareness in this reading measures (Powell et al., 2007; Babayigit and Stainthorp, 2011; Landerl et al., 2019). However, various studies have found that the different components of rapid automatized naming (RAN) might contribute differently to the explanation of different reading measures in different languages and at different ages (Georgiou et al., 2008), which is the reason for distinguishing between alphanumeric (letters and numbers) and non-alphanumeric (objects and colors) components in this study. Thus, it has been found that alphanumeric RAN (aRAN) is more closely correlated to reading than non-alphanumeric RAN (naRAN) due to its relationship with phonological processing (Georgiou et al., 2006, 2008; Aguilar-Villagrán et al., 2010; González-Valenzuela et al., 2016). However, there is no consensus as to whether rapid automatized naming affects children at younger or older ages (Georgiou et al., 2006; Aguilar-Villagrán et al., 2010; González-Seíjas et al., 2013; Kibby et al., 2014), which is why it is considered in this study with children after literacy. Thus, in consistent languages, it appears that rapid automatized naming at an early age is related to reading at a later age (Aguilar-Villagrán et al., 2010; Suárez-Coalla et al., 2013; De la Calle-Cabrera et al., 2019; Torppa et al., 2019), but the connection is weaker among older children (Gómez-Velázquez et al., 2010; González-Valenzuela et al., 2016).

Phonological memory, defined as the capacity for phonological recoding in access to the lexicon and to retrieve phonological information stored in the memory, is needed to learn the sound structure of new words (Nation and Hulme, 2011). In this sense, Gathercole and Pickering (2000) found a significative correlation between phonological memory (non-word repetition) and the literacy at 7 years old. On the other hand, learning to read also influence in phonological memory since it can change the nature of the phonological representations that children have, beginning to incorporate orthographic information (Perre et al., 2009; Pattamadilok et al., 2010). Following this premise, as children learn to read, the phonological representations are restructured, generating greater support for the repetition of non-words in literate people (Nation and Hulme, 2011). It should be noted that this variable has not been widely studied, unlike short-term verbal memory, and when it is studied, the subjects are dyslexic children (Georgiou et al., 2008; Landerl et al., 2013). The few studies that have used this type of variable in normative subjects demonstrate its influence on reading in different languages and in the early years of schooling, without distinguishing variations in the age of children (Gathercole and Pickering, 2000; Georgiou et al., 2008; Suárez-Coalla et al., 2013; Kibby et al., 2014; González-Valenzuela et al., 2016). Most of these studies have found that phonological memory correlates weakly with different reading measures in different languages and at different ages (Gathercole and Pickering, 2000; Georgiou et al., 2008; Mcilraith, 2018). This could be due, on the one hand, to the way of measuring memory, which in most cases it is assayed with verbal memory or digit memory tests, and not with phonological tasks, in which the ability for phonological recoding in the access to the lexicon and the way to recover the phonological information that is stored in the memory are evaluated. On the other hand, it may be due to whether it has been considered jointly with phonological awareness and rapid automatized naming, since there is research supporting that phonological memory only correlates to reading when it is considered together with these variables (Parrila et al., 2004; Suárez-Coalla et al., 2013), due to the relationship between phonological memory and awareness (Wagner et al., 1997). Some of these studies indicate correlation with the reading of words and pseudowords at the age of six in Spanish subjects, somewhat higher in the reading of words (González-Valenzuela et al., 2016). In other consistent orthographies, it has been found to correlate with the reading accuracy of words or pseudowords among older pupils (Georgiou et al., 2008; Binamé and Poncelet, 2016). Other authors argue that it only correlates when considered jointly with phonological awareness and rapid naming (Suárez-Coalla et al., 2013; López-Escribano et al., 2018), due to the relationship between memory and phonological awareness (Binamé and Poncelet, 2016; Verhoeven et al., 2016).

The variety of findings about the contribution made to reading ability by different cognitive variables, such as knowledge of letters, phonological awareness, rapid naming and phonological memory, according to the characteristics of the language and the age of the pupils, and the lack of studies on predicting cognitive factors in reading among older children in Spanish necessitates an analysis of whether the contribution of these variables to the reading ability of Spanish children varies according to age and/or reading experience.

Therefore, the objective of this study is to ascertain the impact made by knowledge of letters, phonological awareness, phonological memory, and alphanumeric and non-alphanumeric rapid automatized naming at the ages of 6 and 7 years on the ability of Spanish children to read words at 7 years of age, when these children are in Year 2 of primary education.

## Materials and Methods

### Participants

As in previous studies (González-Valenzuela et al., 2016), the study sample came from two schools, selected via stratified random sampling, from the school census (CEJA, 2013).

The study included 116 Spanish speaking primary school pupils, 63 boys (54.3%) and 53 girls (45.7%) aged 6 (*M* = 79.74 months, SD = 3.47). Subsequently, when these same children were in Year 2 of Primary Education, they took part in the study again.

17% (*n* = 19) of the fathers and 6% (*n* = 7) of the mothers had a primary level of education; 65% (*n* = 76) of the fathers and 63% (*n* = 73) of the mothers had a secondary education (secondary education, high school, or vocational training); and 18% (*n* = 21) of fathers and 31% (*n* = 36) of mothers had a higher education (degree and post-graduate).

The participating pupils had no intellectual difficulties, no physical or sensory handicaps, or low linguistic status, according to psychological reports compiled by the psychologists of the relevant schools. Pupils originally from other countries who were not fluent in Spanish were not considered.

### Instruments

To evaluate Word Reading (WR), the *Lectura de Palabras* section of the LEE Reading and Writing in Spanish Test (Defior et al., 2006) was used to evaluate fluency in the use of lexical and sub-lexical processes (phonological and orthographic) involved in the reading of words. It consists of reading 42 selected words according to their frequency, length, and spelling complexity. The score of the items in this test depends on the type of reading performed (sounding out, hesitant, fluid). The accuracy of the subjects’ responses for each item is scored between zero and two points. Two points are awarded if the word is read correctly, one point if the word is sounded out and/or read hesitantly, and zero points if the word is read incorrectly. The total WR score was the sum of the scores achieved on each item. The score range for this test is 0–84 points. Cronbach’s alpha statistic (α = 0.93) in Year 2 indicates the reliability of the test.

Knowledge of Letters (KL) was evaluated by means of the *Lectura de Letras* section of the LEE test (Defior et al., 2006), which measures knowledge of all the letters in Spanish. It consists of naming the 29 letters of the Spanish alphabet. The total score is obtained from the sum total of letters correctly named. Cronbach’s alpha statistic for the test carried out in years 1 and 2 was 0.676 and 0.79, respectively.

Another sub-section of the LEE Test (Defior et al., 2006), *Segmentación Fonémica*, which measures phonemic awareness, was used to evaluate Phonological Awareness (PA). It consists of isolating sounds that make up 14 words presented orally, being able to say the name or the sound of the letter. The total score for this is obtained from the sum total of letters correctly named or sounded out. Cronbach’s alpha statistic for the test carried out in years 1 and 2 (α = 0.76 and α = 0.73, respectively) indicates the reliability of the test.

Rapid Automatized Naming (RAN) was evaluated by means of the RAN test (Wolf and Denckla, 2003) adapted by Gómez-Velázquez et al. (2010). The test measures speed in naming visual stimuli. It consists of naming 200 visual stimuli classified equally into letters, numbers, objects, and colors. As in González-Valenzuela et al. (2016), two measures have been considered for this variable: Rapid Naming of Alphanumeric stimuli, letters and numbers (aRAN), and Non-alphanumeric stimuli, objects and colors (naRAN). The total score achieved for these variables is the time taken to name alphanumeric and non-alphanumeric items, respectively. The test-retest reliability of the alphanumeric and non-alphanumeric items was 0.63 and 0.67, for first and second year, respectively.

Phonological memory was assessed using a test of phonological short-term memory developed by Soriano-Ferrer and Miranda (2010), based on Hebrew phonological memory task (Geva et al., 2000). The test requires children to repeat aloud a list of 20 Latin words that are not related to the Spanish lexicon and which do not bear any similarity to Spanish morphology. These words are of different length and are first pronounced by the examiner. The total score for this is obtained from the sum total of pseudowords repeated correctly. Cronbach’s alpha for this test obtained in the described sample was 0.57 and 63, for the first and second years, respectively.

### Procedure

Having obtained authorization from the Experimental Ethics Committee of the University of Málaga (CEUMA) and the parents or legal guardians of the selected pupils, by means of their informed consent, the different instruments were applied individually to each pupil. The tests were administered by four psychologists. When the pupils were in Year 1, they were given tests that evaluated cognitive variables. When they were in Year 2, they were given tests that evaluated these same variables along with the test that measured word reading.

In each year, the instruments were administered over two sessions, each lasting approximately 20 min. In the first session, the tests evaluating the reading of letters and words were administered. In the second session, phonological memory, phonological awareness, and rapid naming (alphanumeric and non-alphanumeric) tests were carried out.

### Statistical Analysis

In accordance with the objective of this study, different types of statistical analyses were performed, descriptive-exploratory, also examining the bivariate relationships between the variables of the study and conducting multivariate regressions for predictive purposes.

Pearson’s correlation coefficients and their corresponding significance tests were calculated to analyze relationships. They were considered small (*r* = | 0.10|), moderate (*r* = | 0.30|) or strong (*r* = | 0.50| or greater) according to Cohen’s (1988) criterion for effect size.

Subsequently, to respond to the main objective of this study, multivariate regressions were modeled to identify: firstly, the cognitive measures at 6 years of age that predict word reading at seven, ascertain the overall contribution of this relationship to its variability, and the individual contribution of each of them; and secondly, the cognitive variables that predict the reading of words at 7 years of age, as well as the global and unique importance of each of them.

The inclusion criteria for independent variables in the construction of these regression models were the statistical and practical significance of Pearson’s *r* statistic, when previous bivariate analyses had an associated probability of less than 0.05 and an effect size equal to or greater than | 0.20|. They were entered sequentially, in decreasing order according to their corresponding correlation coefficient. To evaluate the overall significance of the estimated model and its parameters, Fisher’s *F* test and Student’s *t* test (two-tailed) were used, respectively. The coefficient of determination (*R*^2^) and the adjusted coefficient of determination (Adjusted*R*^2^) were used to assess overall the variance of word reading attributable to the regression model. To specify the contribution of each predictor to the total variance of word reading, the semi-partial correlation coefficient ($s\,r_{i}^{2}$) was used. The practical significance of the estimated final regression models was evaluated using the measure of effect size from Cohen’s (1988) family of correlations *f*^2^, used for the multiple regression, with reference values being small = 0.02; medium = 0.15 and large = 0.35. The assumptions of the regression models (linearity, normality, and homogeneity of variances) were verified *a posteriori* by means of the analysis of residuals. The variance inflation factor (VIF) was used to test the assumption of multicollinearity; values higher than 10 would indicate a high degree of multicollinearity.

Statistical data processing and analysis were carried out using version 23 of the Statistical Package for the Social Sciences (SPSS).

## Results

Table 1 summarizes the descriptive and bivariate analyses for the variables studied, together with their corresponding statistical significance.

**TABLE 1:** Descriptive statistics and correlations between reading words and the cognitive variables measured at 6 and 7 years of age.

***Variables***	***Mean***	***SD***	***Range***	**1**	**2**	**3**	**4**	**5**	**6**	**7**	**8**	**9**	**10**
**Six years of age**													
1. KL	25.98	2.42	18–29	–									
2. PA	8.34	2.40	1–14	0.37**	–								
3. PM	16.43	2.33	10–20	0.19*	0.27**	–							
4. aRAN	74.17	14.60	45–126	–0.03	–0.09	0.08	–						
5. naRAN	179.37	41.43	91–333	−0.21*	–0.17	–0.18	0.30**	–					
**Seven years of age**													
6. KL	27.01	1.50	22–29	0.56**	0.30**	0.25**	0.02	–0.08	–				
7. PA	10.15	2.39	1–14	0.45**	0.48**	0.22*	–0.17	−0.31**	0.34**	–			
8. PM	16.76	2.33	8–20	0.11	0.03	0.33**	0.10	–0.10	0.19*	0.03	–		
9. aRAN	62.79	12.73	42–103	–0.16	–0.13	0.09	0.58**	0.33**	0.02	−0.21*	–0.01	–	
10. naRAN	154.11	37.17	89–290	−0.25**	–0.17	–0.09	0.21*	0.65**	–0.05	−0.29**	–0.02	0.35**	–
11. WR	67.17	7.40	44–81	0.39**	0.37**	0.32**	–0.15	−0.21*	0.29**	0.38**	0.21*	−0.21*	-0.13

** Pearson’s correlation coefficient *r* significant at <0.01; * Pearson correlation coefficient *r* significant at *p* < 0.05.

*Correlation coefficient effect size *r* (Cohen reference values: Small = | 0.10|; moderate = | 0.30|; strong = | 0.50| or greater).*

*SD, standard deviation; KL, knowledge of letters; PK, phonological awareness (no. of correct answers); PM, phonological memory (no. of correct answers); aRAN, alphanumeric rapid automatized naming (seconds); naRAN, non-alphanumeric rapid automatized naming (seconds); WR, Word reading (fluency).*

The associations between all cognitive measures and word reading were mostly statistically significant. The statistically significant correlations found between the cognitive variables measured at 6 years of age, when students were in Year 1 of primary school, and the reading of words measured at the age of seven, when in Year 2, were knowledge of letters (*r* = 0.39, *p* < 0.01), phonological awareness (*r* = 0.37, *p* < 0.01), phonological memory (*r* = 0.32, *p* < 0.01), and non-alphanumeric rapid naming (*r* = −0.21, *p* < 0.05). No relationship was found with alphanumeric rapid naming (*r* = −0.15, *p* = 0.11).

When the students were 7 years old, with all the variables measured at the same time, in decreasing order according to the size of the correlation, a relationship was found between the variables phonological awareness (*r* = 0.38, *p* < 0.01), knowledge of letters (*r* = 0.29, *p* < 0.01), phonological memory (*r* = 0.21, *p* < 0.05), and alphanumeric rapid naming (*r* = −0.21, *p* < 0.05) and word reading. No statistically significant relationship was found with non-alphanumeric rapid naming (*r* = −0.13, *p* = 0.15).

The results obtained in the regression analysis for word reading in Year 2 of primary school are summarized below (Tables 2, 3).

**TABLE 2:** Regression analysis results for word reading at 7 years of age from cognitive variables at 6 years of age.

**Predictors at the age of six**	***B***	***SE***	**β**	***t***	***p***	***sr***	**VIF**
Constant	30.42	7.27		4.18	0.000		
KL	0.82	0.27	0.27	3.03	0.003	0.25	1.17
PM	0.64	0.26	0.21	2.43	0.017	0.20	1.09
PA	0.58	0.25	0.21	2.35	0.021	0.19	1.22
**Goodness-of-fit tests**							
*F*	12.37**						
*R*^2^	0.25						
*R*^2^ *adjusted*	0.23						
*R*	0.50						

** Fisher’s *F* test significant at *p* < 0.01.

*KL, knowledge of letters; PM, phonological memory; PA, phonological awareness; SE, standard error; sr, semi-partial correlation; VIF, variance inflation factor.*

**TABLE 3:** Regression analysis results for word reading at 7 years of age.

**Predictors at the age of seven**	***B***	***SE***	**β**	***t***	***p***	***sr***	**VIF**
Constant	45.00	5.21		8.63	0.000		
PA	1.16	0.26	0.37	4.40	0.000	0.37	1.00
PM	0.62	0.27	0.19	2.30	0.023	0.19	1.00
**Goodness-of-fit tests**							
*F*	12.67**						
*R*^2^	0.18						
*R*^2^ *adjusted*	0.17						
*R*	0.43						

** Fisher’s *F* test significant at *p* < 0.01.

*PA, phonological awareness; PM, phonological memory; SE, standard error; *sr*, semi-partial correlation; VIF, variance inflation factor.*

Firstly, with word reading measured at the age of seven as a dependent variable and cognitive variables measured at the age of six as the independent variables, the final adjusted model [*F*(3,112) = 12.37, *p* < 0.001, *f^2^* = 0.33] included the variables knowledge of letters [*t*(115) = 3.03, *p* < 0.01], phonological memory [*t*(115) = 2.43, *p* < 0.05], and phonological awareness [*t*(115) = 2.35, *p* < 0.05], explaining 25% (23% adjusted) of variance in the response variable in Year 2 (*R*^2^ = 0.25). Their corresponding semi-partial correlation coefficients ($s\,r_{i}^{2}$) indicated that these three initial cognitive variables contributed, respectively, 6.25, 4, and 3.61% to total variance in word reading when the pupils were 7 years old.

Secondly, we examined which cognitive skills at the age of seven predicted word reading, measured at the same age. The analysis procedure resulted in a final model with good overall fit [*F*(2,113) = 12.67, *p* < 0.001, *f^2^* = 0.22], which included the cognitive variables phonological awareness [*t*(115) = 4.40, *p* < 0.001] and phonological memory [*t*(115) = 2.30, *p* < 0.05]. The coefficient of determination (*R*^2^ = 0.18) indicated that these variables as a whole explained 18% (17% adjusted) of variability in word reading, measured in Year 2. Their corresponding semi-partial correlation coefficients ($s\,r_{i}^{2}$) indicated that the unique contribution of the first one was 13.70% and of the second was 3.61%.

Analysis of residuals and variance inflation verified that these models fit the assumptions of linear regression.

## Discussion

The objective of this study was to ascertain the impact made by certain cognitive variables (knowledge of letters, phonological awareness, phonological memory, and alphanumeric and non-alphanumeric rapid automatized naming) at the ages of 6 and 7 years on the ability of Spanish children to read words at 7 years of age, when these children are in Year 2 of primary education.

The cognitive variables measured at 6 years of age that predict performance in word reading at the age of seven, when pupils are in Year 2 of primary school, are knowledge of letters, phonological memory, and phonological awareness. Knowledge of letters at 6 years of age is presented as the initial predictor that explains the highest level of variance in word reading at 7 years of age. The other variables explain similar percentages of variance. Neither alphanumeric nor non-alphanumeric rapid automatized naming were part of the estimated final regression model.

At 7 years of age, it has been found that, of all the cognitive variables measured at that age, phonological awareness, and phonological memory are that ones that significantly explain the reading of words at that time in their learning, explaining a considerable substantive magnitude of their variability. Of these, phonological awareness explained a much greater degree of variance. Neither knowledge of letters nor alphanumeric/non-alphanumeric rapid automatized naming were part of the estimated final regression model.

In general, the findings show that the cognitive variables considered at 6 and 7 years of age contribute differently to the explanation of reading at 7 years of age, coinciding with other studies conducted in different languages and at different ages. That is, not all the cognitive variables considered contribute equally to the explanation of word reading, and this contribution also varies with age (Georgiou et al., 2006; Li et al., 2012; Caravolas et al., 2013; González-Valenzuela et al., 2016; Asadi et al., 2017; López-Escribano et al., 2018; Mcilraith, 2018; Bar-Kochva and Nevo, 2019; Landerl et al., 2019; Torppa et al., 2019; Vibulpatanavong and Evans, 2019; Wijaythilake et al., 2019). In particular, word reading in Year 2 (7 years of age) is explained by knowledge of letters, phonological awareness, and phonological memory when the children are 6 years old, while it is only explained by phonological awareness and phonological memory when the children are 7 years old. Alphanumeric and non-alphanumeric rapid naming do not contribute to the explanation of reading ability in Year 2.

According to these results, in Spanish, knowledge of letters is important in reading words at slightly older ages, as in other languages (Casillas and Goikoetxea, 2007; Caravolas et al., 2012, 2013; Georgiou et al., 2012; De la Calle-Cabrera et al., 2019; Wijaythilake et al., 2019), but it stops being a factor as age increases (Landerl et al., 2013; Solari et al., 2014; Paige et al., 2018). This may be because, in Spanish, at older ages, knowledge of letters would already be acquired in normative subjects and would no longer have relevance in the reading of words.

In addition, it is also found that phonological awareness and phonological memory are important in the reading of words in Spanish and that they maintain their contribution in Year 2. These results coincide with those of other studies indicating that, when the letters are already known, the important thing is to learn to assemble them in words through the establishment of the grapheme-phoneme correspondence, and the retention of verbal stimuli is necessary (Georgiou et al., 2008; Furnes and Samuelsson, 2010; Li et al., 2012; Caravolas et al., 2013; González-Seíjas et al., 2013; Suárez-Coalla et al., 2013; Kibby et al., 2014; González-Valenzuela et al., 2016; Bar-Kochva and Nevo, 2019).

On the one hand, these results highlight the importance of phonological awareness even at reading fluency measures and at slightly older ages in consistent languages such as Spanish (Casillas and Goikoetxea, 2007; Georgiou et al., 2008; Furnes and Samuelsson, 2010; Li et al., 2012; González-Trujillo et al., 2014; González-Valenzuela et al., 2016; Bar-Kochva and Nevo, 2019). The effect of sound-letter correspondence and phonetic instruction in orthographically consistent languages is powerful enough to ensure children’s phonological recoding skills after they have already gained some reading experience (Georgiou et al., 2008, 2012; Caravolas et al., 2013; Landerl et al., 2013; Kibby et al., 2014). In addition, the difficulty of the task used to measure phonological awareness (isolating sounds) might also explain the influence of phonological awareness on word reading, as some studies indicate (Caravolas et al., 2013; Vibulpatanavong and Evans, 2019).

On the other hand, the contribution of phonological memory has also been seen to be important in reading Spanish words at the age of seven, in accordance with some studies (Gathercole and Pickering, 2000; Nation and Hulme, 2011). In this sense, it has been found that the phonological recoding in the access of the lexicon and the recovery of the phonological information that is stored in the memory are important in order to learn the sound structure of new words. Nevertheless, it seems that the predictive power of the phonological memory appears to be less than that of phonological awareness, as indicated by other studies (Georgiou et al., 2008; Binamé and Poncelet, 2016; Mcilraith, 2018). Most of these studies highlight the importance of phonological awareness versus the other cognitive variables considered at different ages, as it would provide tools to establish the corresponding relationships between graphemes and phonemes. The contribution of phonological memory might be due to the fact that it has been considered in conjunction with phonological awareness and rapid automatized naming, favoring the acquisition of the grapheme-phoneme conversion rules (Suárez-Coalla et al., 2013) and the relationship between memory and phonological awareness (Binamé and Poncelet, 2016).

Finally, studies support the influence of rapid automatized naming on reading in alphabetical languages (Georgiou et al., 2008, 2016; Aguilar-Villagrán et al., 2010; Furnes and Samuelsson, 2010; Ferroni et al., 2016; González-Valenzuela et al., 2016; López-Escribano et al., 2018; Bar-Kochva and Nevo, 2019; De la Calle-Cabrera et al., 2019), since the reader decodes by applying the grapheme-phoneme conversion rules and the phonological representation of each grapheme must be recovered quickly for the grapheme-phoneme recoding strategy to be effective. However, this study has found that rapid automatized naming does not appear to influence the reading of Spanish words, measured as fluency, at 7 years of age, despite distinguishing between alphanumeric and non-alphanumeric components.

On the one hand, these results are in line with studies that argue that non-alphanumeric rapid naming does not contribute to explaining reading in subjects of different languages (Aguilar-Villagrán et al., 2010; González-Valenzuela et al., 2016). This may be due to the nature of the non-alphanumeric items themselves, which do not have specific linguistic content, and therefore at these ages it would no longer have relevance in reading (Georgiou et al., 2008; Suárez-Coalla et al., 2013). Non-alphanumeric RAN appears to be more related to general verbal processing, as opposed to alphanumeric RAN, which would be more related to more specific processing, phonological processing (Georgiou et al., 2008). Furthermore, the importance of these non-alphanumeric components appears to be different according to age and linguistic characteristics (Aguilar-Villagrán et al., 2010; Li et al., 2012; Suárez-Coalla et al., 2013). In some of these studies, children were pre-readers (Aguilar-Villagrán et al., 2010; Suárez-Coalla et al., 2013) and in other cases they were speakers of more inconsistent languages (Li et al., 2012; Landerl et al., 2013).

On the other hand, there are studies that indicate that alphanumeric rapid automatized naming at older ages does not seem to be so strongly related to the reading of words (Gómez-Velázquez et al., 2010; González-Valenzuela et al., 2016). This might happen because at these ages the phonological representation of each grapheme is recovered quickly due to the literacy level that the children have already acquired. Some studies also suggest that the relationship found between aRAN and reading is mediated by the influence of phonological awareness (Bar-Kochva and Nevo, 2019), becoming less important in reading when considered together, and that it depends on the relationship of independence or dependence between them (Casillas and Goikoetxea, 2007; González-Seíjas et al., 2013). In this sense, some studies argue that rapid naming predicts word reading to a lesser extent than phonological awareness, depending on age (González-Seíjas et al., 2013; Suárez-Coalla et al., 2013) and spelling consistency (Georgiou et al., 2008; Furnes and Samuelsson, 2010; Landerl et al., 2013).

Based on the results found, analysis of the relationships between the cognitive variables at 6 and 7 years of age would have been useful to explain their contribution to reading after literacy. Further research in different languages and at different ages addressing the relationship between the cognitive variables considered would be necessary to better understand the degree of dependence or independence between them and how this may influence their relationship with reading in post-readers. In subsequent studies, an analysis of these relationships and a comparison of results in Spanish with less consistent languages at older ages will be performed, in order to analyze whether linguistic characteristics and reading experience influence these relationships once the process of teaching-learning the written language has begun. It would also be convenient in future studies to analyze the impact of the variables studied together with more classic cognitive variable, such as vocabulary, morphology, and syntax, on the reading of words in Spanish, in line with other studies performed in other languages (Schechter et al., 2018).

Finally, it should be noted that these results could have important educational implications in terms of preventing difficulties in the learning of reading in Primary Education, as it highlights the importance of cognitive variables compared to others when it comes to addressing literacy in normative subjects. The findings show how phonological awareness remains a strong component in the learning of reading in Spanish even at slightly older ages.

## Data Availability Statement

The raw data supporting the conclusions of this article will be made available by the authors, without undue reservation.

## Ethics Statement

The studies involving human participants were reviewed and approved by the Comité Ético de Experimentación de la Universidad de Málaga (CEUMA). Written informed consent to participate in this study was provided by the participants’ legal guardian/next of kin.

## Author Contributions

All authors listed have made a substantial, direct and intellectual contribution to the work, and approved it for publication.

## Conflict of Interest

The authors declare that the research was conducted in the absence of any commercial or financial relationships that could be construed as a potential conflict of interest.
